# The role of digital health in the cardiovascular learning healthcare system

**DOI:** 10.3389/fcvm.2022.1008575

**Published:** 2022-11-03

**Authors:** Ragasnehith Maddula, James MacLeod, Tyson McLeish, Sabrina Painter, Austin Steward, Generika Berman, Abdulaziz Hamid, Mohamed Abdelrahim, Jeffrey Whittle, Sherry Ann Brown

**Affiliations:** ^1^Medical College of Wisconsin, Milwaukee, WI, United States; ^2^Zilber School of Public Health, University of Wisconsin-Milwaukee, Milwaukee, WI, United States; ^3^Medical College of Wisconsin, Green Bay, WI, United States; ^4^Division of Internal Medicine, Medical College of Wisconsin, Milwaukee, WI, United States; ^5^Cardio-Oncology Program, Division of Cardiovascular Medicine, Medical College of Wisconsin, Milwaukee, WI, United States

**Keywords:** digital health, digital medicine, health innovation, learning healthcare system, artificial intelligence, cardio-oncology, wearables, health technology

## Introduction

Modern medicine has undergone immense transformation in the past decade with the discovery, innovation, and development of novel health systems and advanced patient care brought forth by technological progress. Synchronously, developments in technology have created what many call, a “paradigm shift” in the way society interacts with technology, as well as the impact and ubiquity of technology in our livelihood (3). This transformation is influencing medicine and modern science in many aspects. One example is the adoption of digital health which includes “disruptive technologies that provide digital and objective data accessible to both caregivers and patients” (3); according to the Food and Drug Administration (FDA) these disruptive technologies include mobile health (mHealth), health information technology, wearable devices, telehealth and telemedicine and personalized medicine (2).

Digital health represents an important aspect of health for the future, and when applied to medical practice, can be termed digital medicine. Initiatives in digital medicine are leading healthcare and traditional models of medicine to evolve and address the changing dynamic of patient-physician relationships and overall clinical outcomes (4). In digital medicine, hardware and software tools power technology that supports the practice of medicine, such as disease prevention and treatment for individuals and populations. While electronic health record (EHR), registry, and claims data will predominate in the near term, they cannot provide a complete picture of the various factors that influence a patient’s cardiac health. As a result, technologies that can provide an accurate account from outside of hospitals and clinics will become more important in the cardiovascular learning healthcare system (2). As mentioned by an American Heart Association (AHA) Scientific Statement, the absence of defined procedures for analysis and application of the clinical uses of digital health technologies remains a large hurdle (5). This problem is being addressed, however, as the American College of Cardiology (6) and the FDA (2, 7) have recently released preliminary guidelines on this topic. The process of amassing relevant data to further shape guidelines will take time.

This review explores the role of digital health and how we can maximize benefits for patients and the health system in general in the context of a learning healthcare system (LHS). The Agency for Healthcare Research and Quality defines a LHS as one in which observational data generated within the system are synthesized with scientific evidence from outside the system to provide patients with safer, more efficient, and higher quality healthcare (1). The AHA released a scientific statement on LHS in 2017 (5), which includes how health information technology and health data can be leveraged to ensure that “evidence informs practice and practice informs evidence” (8). This integration includes high quality data from the literature which is then woven into routine practice. Dissemination of evidence-based information and responsiveness to feedback also allows LHS usage to improve work environments for employees (1). In a LHS, the component technologies of digital health are outlined and contextualized to illustrate their impact on enabling patients to understand and visualize medical prognosis in a user-friendly manner (9). By understanding the motive, expectations, and development of digital health, we will better understand the direction in which disruptive technologies continue to revolutionize medicine (3).

Recent events have amplified the necessity of digital and connected health offerings. The coronavirus disease of 2019 (COVID-19) has accelerated accessibility, adoption, and efficacy of these offerings (10). However, barriers still exist in assessing these technologies and ensuring integration is done in an equitable way. Working with industry to conduct robust studies on clinical efficacy of various products can give confidence to health care providers recommending their use and will begin to build a body of evidence to continue the growth of insurance coverage for digital health. In an effort to contribute to and guide the growth of this body of evidence, this review outlines the role of digital health in the LHS, delineates challenges in system implementation and notes considerations that should be made to ensure equitable, and patient centered integration into the pre-existing system.

## Digital health in the pandemic

The COVID-19 pandemic caused a rapid shift toward telemedicine, mHealth and digital health due to restrictions made on elective procedures and regular clinics visits (11). This paved the way for physicians, health care providers, and patients to maintain communication safely through digital means (11, 12). The scope of digital health includes technologies in the form of mobile applications, eHealth, and wearables [e.g., Electrocardiogram (ECG) monitors, blood pressure sensors] to health diaries and instructional videos for patient care. The facilities and infrastructure that have used digital health in the pandemic have consequentially benefited large patient populations considered immunocompromised or at-risk (13). Some hospital systems took initiative to reduce potential exposures and transmission by integrating artificial intelligence (AI) into their pre-hospital triage procedures, including employing an AI-based COVID-19 screener tool used to assess patient risk and lower the volume of abandoned calls on their COVID-19 hotline (14). These tasks were traditionally performed by clinical staff and the transition to AI allowed for a reduction in the consumption of resources.

In 2021, the use and value of remote patient monitoring (RPM) through wearables to enhance virtual patient care had accelerated to protect individuals from exposure and continue providing optimal care and monitoring (14). RPM provides patient data to clinicians outside the healthcare facility, which is essential because continuous access to real-time physiological data improves physician oversight of patient health. The use of RPM has accelerated since COVID-19 started, from 7 million patients using RPM in 2016 to over 23 million in 2020 (15). The number of patients utilizing remote health monitoring tools is estimated to increase to 30 million by 2024 (16). In cardiology, digital health has become an immensely growing component in transforming cardiac medicine. There are many benefits to digital medicine. However, there are challenges that will need to be addressed for digital medicine to fulfill its potential. Such challenges include adoption and implementation especially regarding under-resourced populations potentially being left behind (15).

Through the lens of the ongoing global pandemic, telemedicine has provided adequate medical support to thousands of patients. Telemedicine increases access to healthcare for patients across communities and can provide a cheaper alternative to modern healthcare when physical visitations may not be required for all patients. Combined with lowered costs, for both patients and the healthcare system, telemedicine a category of digital health, provides an efficient alternative for non-emergency situations, while also expanding the reach to underserved communities that may not have accessible facilities for healthcare nearby (16, 17). By bridging the gap between quality health care and populations in need, clinical outcomes and overall health in patients can improve through many forms of digital health (18).

## Need for digital health

While there was a robust expansion in the usage of digital health technologies during the pandemic to expand access to healthcare, there are other utilities for digital health. The usage of digital health is commonly associated with increased access to healthcare. However, digital health is needed in a LHS to enhance the clinician-patient relationship in several ways. Digital health interventions promote effective clinical care. An example of this is the CardioMEMs system. In the CardioMEMs system (St. Jude Medical, St. Paul, MN), cardiac catheterization is used to permanently implant a “wearable” pressure sensor in the pulmonary artery that communicates with an external data collection device to send pulmonary artery pressure, pressure waveforms, and heart rate data to a secure cloud-based website, allowing early detection of worsening heart failure (5, 19). In initial clinical trials, patients with New York Heart Association class III heart failure who received the device experienced a 37% reduction (*P* < 0.0001) in heart failure hospitalizations over a 15-month mean follow-up period (5, 19). This system is one of the most successful and early applications of digital health in cardiology. Digital health interventions within cardiovascular care have been crucial in assessing the effect of health technologies in improving patient health self-management and outcomes, with one particular study observing patients with acute myocardial infarctions presenting with a predominantly higher level of patient activation in self-management as well as fewer 30-day readmissions (20). Other digital health interventions have been implemented and studied for several cardiovascular health applications, such as heart failure diagnosis and management, risk assessment, cardiac rehabilitation, and peripheral vascular disease management, with promising results (21). Digital health tools have the capability to attenuate risk factors throughout disease processes such as cancer, during prehabilitation, habilitation, and rehabilitation (22). Physicians in many fields are increasingly considering implementing digital health solutions into their practice. The AMA “Physicians’ Motivations and Key Requirements for Adopting Digital Health Adoption and attitudinal shifts from 2016 to 2022” study outlines improving health outcomes, work efficiency, and diagnostic ability as key drivers for physicians considering implementing digital health into their practice (23). Notably, about 3 in 5 physicians say technology can help address key needs with chronic disease patients, preventative care and automating administrative tasks (23).

Incorporating digital health tools into a LHS will lead to stronger patient-physician relationships and increased personalization of care, as patients can transmit RPM and mHealth data which provides insight into the day-to-day factors that influence cardiovascular health and disease. In addition, when patients use digital health tools to transmit real-time, objective clinical and subjective data, they are empowered because they are more involved in their care and the decisions their providers make (5). For example, patient portals empower patients with self-service functions such as appointment scheduling, secure messaging with providers, access to test results, and personal health information. In addition, patient portals are beginning to integrate with digital platforms that provide RPM-centered functions, establishing infrastructure for alert and referral systems based on vital signs, biomarker tracking and other critical biometrics (23, 24). This change has enhanced the patient-physician relationship by streamlining workflow and allowing patients and providers to rapidly establish lines of communication with one another when there are changes in a patient’s condition. An example of this combined digital health workflow is a patient using a blood pressure device and uploading their results to an integrated patient portal digital platform. The patient’s physician can review this information, and communication about the results can occur *via* secure patient portal messaging or a scheduled telemedicine visit. For patients with an increased risk for severe COVID illness, all aspects of care can be addressed through a digital health-centered workflow without the patient having to leave the safety of their own home (23, 24). Trust is fostered with more regular interactions between patients and providers, including the use of secure messaging, patient portals, mHealth apps, and other digital health tools.

There is a need for healthcare providers to manage an incredible amount of clinical information as healthcare delivery systems become increasingly more complex. An increased demand for clinicians to manage these clinical data can lead to inconsistencies between data reporting between providers or health systems, uninterpretable data, or missing data (24). The LHS will benefit from the use of AI-based digital health tools that integrate EHR across practices to provide structure to EHR data that are otherwise organized on a practice-to-practice basis. AI tools also provide us with a means to extract actionable clinical data that is buried amongst irrelevant clinical data. As the AHA LHS statement outlines, collaboration of medicine and technology will be a necessary foundation in the future to improve the quality, access, and effectiveness of patient care through technological advances. A LHS integrates and evolves the current healthcare system to use health data to apply scientific discovery at the point of care and uses insights from said clinical care to inform future care (5). Further, as efficiency is optimized, digital health can improve cost and utilization in healthcare. For example, the CardioMEMS heart failure system was found to be cost effective compared with the standard of care treatment (25). Additionally, a study analyzing healthcare utilization associated with digital health intervention in asthma treatment demonstrated a reduction in hospitalizations and emergency department visits (26). Integration of digital technologies into existing EHR systems may increase productivity and cost-effectiveness of these systems by enhancing existing EHR strengths in chronic and medical care management and communication efficiency between and within organizations (27). Limited healthcare resources necessitates advancements that create better health outcomes at a lower cost; The described digital health technologies show that these goals are achievable.

## Vast range of potential technologies

**FIGURE 1:**
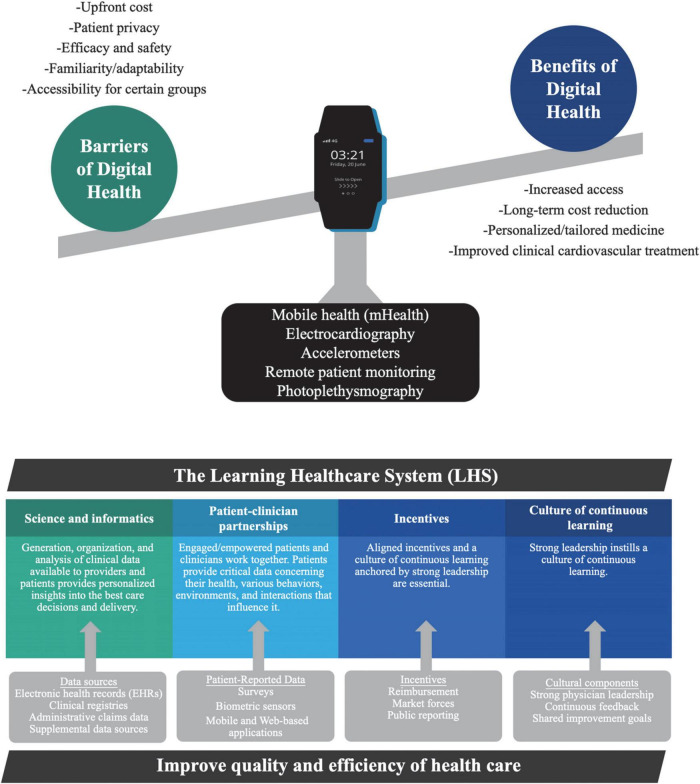
Digital health architecture and pillars of the learning healthcare system. The digital health architecture provides clinicians with insight into patients’ health and lifestyle outside of healthcare settings. Patient-reported data is recorded with: mobile health (mHealth), biometric sensors (including wearables), and web-based applications. The four key components of the learning healthcare system are data sources, patient-reported surveys, incentives, and cultural components to bolster novel findings and improve quality of care. Templates from Infograpia were used in these graphics.

Improvement in healthcare practices is predicated on the integration of technology through all aspects of medicine. We are embarking on an era of digital medicine that has enabled progress in patient care due to the ability of technology to reduce costs, improve access, collect data and personalize medicine for patients (28). Wearables (e.g., ECG monitors, blood pressure sensors, etc.), digital health diaries, and electronic instructional modules have started to transform how we deal with disease by improving management and maintenance (29). Mobile applications can increase access to various demographics and integrate mobile health into regular clinical practice (Figure 1) (30–32). The increased integration of technology in medicine not only stems from the consistent advancements in technology but also from the growing scope of practices that can be improved. The full potential of digital medicine is still unexplored due to the vast range of available technologies. Initially, technology integration in healthcare focused on patient monitoring and charting to optimize patient care. Digital technology is now involved in numerous sectors of medicine, including diagnostics, health information technology, mobile health, telemedicine, and wearable devices (5). Digital health interfaces with or includes variations of telemedicine and biometric tracking, as well as digital applications that diagnose, augment treatment, and increase access to resources for medical conditions, especially those related to preventive care and mental health (7, 28). The expounding potential of medicine stems from personalized care, where patient data, genetic testing and wearable devices can create individualized treatment plans to cater to unique needs. Technology that categorizes and captures the characteristics of patient populations, specifically high-cost patients, can direct allocation of resources and tailor interventions to optimize care (33). The US Institute of Medicine has two imperatives that each kind of health focused technology must address. The first requires technology to be informational and the second requires technology to provide value (34). Being informational is defined as helping providers and patients navigate the increased scientific body of knowledge and complexity of the medical system. The technology or digitization can contribute to the second imperative value, where there is a lack of cost transparency or a mismatch of incentives. An example of a company that claims to fit both imperatives has developed technology to simplify healthcare plans for seniors by using software to guide physician recommendations based on what is covered by Medicare and other insurance plans. The technology empowers providers with AI-driven personalized insights at the point of care.

The vast range of potential technologies is evident by the smart wearable products available on the market today. Each device collects distinct biological measurements that are conducive to various cardiovascular clinical applications. Wearable activity sensors are advantageous to clinicians to provide accurate daily data of patient physical activity levels as an active lifestyle is a critical component of promoting cardiovascular health. This can be accomplished through data capture by accelerometers, Global Positioning System (GPS) devices or barometers. Accelerometers measure the linear acceleration of movement along triaxial planes, which is useful for tracking step count, speed, and sedentary time (21). GPS can coordinate distance traveled while barometers can measure the stair count of exercise. Wearable heart rate and rhythm sensors are pragmatic approaches to detecting daily hemodynamic changes in patients for clinical monitoring through ECG or photoplethysmography sensors. ECG sensors track cardiac electrical activity and monitor patients for potential electrolyte abnormalities and arrhythmias. Photoplethysmography sensors can evaluate heart rate, blood pressure (BP) and cardiac output through measurement of changes in microvascular blood volume. BP sensors are necessary to predict, monitor, and track the potential for the development and progression of hypertension by providing clinicians with comprehensive tools for treatment. An example of this is an oscillometer, which is a blood pressure measurement device worn on the wrist that displays readings through a smartwatch monitor. The use of various sensors has diversified wearable approaches, as they can be worn through clothing and shoe embedded sensors, smartwatches, smart bands, chest straps, ECG patches and medical earbuds (21).

Another frontier of technology that manifests promise in the field of digital health is the rise of AI-based applications, especially in the field of cardiology. Advances in deep learning aspects of AI-based systems have propelled the use of this technology in clinical cardiovascular treatment. AI, and more specifically, precision medicine, have been used in concordance with clinicians to provide advanced frameworks for developing cardiovascular therapeutics through alternative approaches to cardiovascular risk stratification and phenotyping heart failure (35). AI has been integral in maximizing the efficiency of association studies, developing the expansion of precision medicine and the potential to improve patient care through this novel framework of capabilities (35). The AHA statement on learning healthcare systems highlights the utility of predictive analytics in understanding patient and environmental data to maximize benefits in individual and population health, in conjunction with EHRs (5). Beyond integrating commercial technologies, applications in which predictive analytics would be useful in a LHS include evaluating high-cost patient care, anticipating readmissions, predicting adverse events and projecting the trajectory of diseases, further enhancing the integration between digital technologies and healthcare systems in improving modern medicine (5). An example of this is mobile health (mHealth), which includes the use of newly developed smartphone-connected applications in resource-limited areas to assist in diagnosing rheumatic and heart diseases (36). A trial of this technology in mHealth clinics in India demonstrated that patients randomized to mHealth diagnostic assessments were associated with a lower risk of hospitalization and/or death on follow-up (15% vs. 28%) (36). This substantiates the potential and necessity for digital health within clinical frameworks and in providing optimal patient care. The most important component to successful digital health integration is adherence from not only clinicians but patients as well. The exponential advancement and integration of technology in all parts of our lives has strengthened openness of Americans to digital health integration. The positive perceptions of digital health among Americans can be shown by 50% of American adults switching from in-person to telehealth appointments in the past year and the growth of 7 million patients in 2016 using remote devices for monitoring their health to over 23 million users in 2020 (37). Overall, the integration of a vast array of technologies in health care is not a possibility but an inevitability, which provides a positive outlook for the future of healthcare.

## Digital health for patient education and engagement

Patient education and medical literacy have steadily improved since the influx of disruptive technology in recent decades. This is in part due to the immense troves of information available on the internet, as well as applications and databases that have allowed patients to understand more colloquially medical terminology, prognosis, and treatment options (3, 38). With increased accessibility to medical information online, modern medicine has had to adapt to the increase in curiosity and need for clarity for patients seeking better understanding of their health and complications (39). Patient education has taken many forms, such as education on nutrition, health hygiene, and maintaining healthy habits, or on methods for tracking personal biometrics to understand one’s health. Innovative and interactive technologies, such as ECG wearables, health diaries, telemonitoring, blood pressure sensors, and several others have expanded the scope of health management, for patients as well as healthcare professionals. The process of incorporating digital health technologies into daily practice among providers and patients is gradually becoming commonplace (18, 38). Several studies have evaluated the impacts of integrating digital health technologies as a method of patient education and engagement in their health management, particularly with medication adherence, health practices and improving clinical or laboratory outcomes. Voice recognition technologies used in the medical management of patients with chronic heart failure showed a potential to better control sodium intake, improved Minnesota Living with Heart Failure (MLHFQ) scores, as well as greater quality of life, highlighting the importance of self-management in the long term prognosis of cardiovascular disease (40). Medication adherence and lifestyle modifications, which have a critical role in cardiovascular disease management, were shown to be positively influenced by mobile phone-based interventions through short messaging services in addition to virtual training, face-to-face counseling, electronic pillboxes and home monitors (41, 42). This natural evolution has increased the visibility of health information, substantially increasing the decision-making power of patients regarding their own health, as opposed to the traditional model of medicine heavily reliant on physician responsibility.

Digital health has also begun to incorporate advanced technologies in AI in various aspects of medicine, importantly in understanding biomarker progression in patients, and establishing a system that expedites the referral process and mitigates emergency situations when they do arise (18). Immediate alerts and telemonitoring subsequently improve overall health outcomes as physician, and patient oversight is increased, along with understanding of biodata and evaluating paths of treatment and intervention (42).

The prevalence of digital health today can also be attributed to the improvements and innovations in user experience with health technologies. With improved user interfaces, patients are now able to quickly understand how the technology works to facilitate incorporation into their daily lives. Adherence to medications and treatment plans have also been improved, as trackers and sensors, along with app monitoring and reminders have improved patient adherence, while also allowing providers to maintain oversight. This component of medicine has always been difficult to oversee, as patients may not always keep track of their medications and dosage intervals, leading to reduced adherence and worse outcomes. For example, in individuals with asthma, sensors have been incorporated into inhalers to track inhaler usage, and also to determine location of patients to understand environmental triggers and factors leading to asthma exacerbation (43). These applications improve the quality of care, help providers understand factors that trigger negative responses, and further improve the healthcare process by treating patients using multifaceted measures and approaches. Insights suggested by health technologies contribute to the overall transformation of healthcare by introducing new parameters and perspectives not previously incorporated in medicine. By doing so, providers and patients can take better measures for interventions, and preemptive measures to reduce future complications.

## Assessing health technologies

While digital health technologies have already begun changing lives for the better, there are many, often overlooked, pitfalls of apps and technologies. Proper evaluation of a technology’s utility and clinical impact is necessary to ensure that clinical care and patient well-being are not compromised in the name of convenience, higher billings, or expediting clinical workflow. Data breaches, false measurements and assessments, and exacerbation of the very health issues being treated are all potential side effects (44, 45). While we amass clinical data regarding new health technologies, patterns of risks and benefits will become clearer as well as which technologies are most efficacious. We are starting to understand the benefits of these technologies but there remains a large gap between their development and precise evidence-based implementation into clinical practice (46). Just like with any medication, we must first prove the efficacy of a new technology, act directly to minimize harm potential and ensure that there are no more beneficial standards of care in place. Only then should a physician feel comfortable prescribing or recommending an application, software, or hardware device to their patient. Regulation of digital health technologies is carried out by the FDA and is an evolving process. Their Digital Health Center of Excellence marks the beginning of a comprehensive digital health approach providing regulatory guidance to digital health companies and education for stakeholders through a pool of digital health resources (2). They also have released draft guidance documents for the use of remote patient monitoring with digital health technologies. Their guidance document, Digital Health Technologies for Remote Data Acquisition in Clinical Investigations released in January 2022, provides non-binding recommendations on the use of digital health technologies to acquire clinical investigation data remotely (7). This document will help direct the research necessary for physician guidance on implementing digital health. Beyond the FDA, the American College of Cardiology’s Best Practices for Consumer Cardiovascular Technology Solutions framework emphasizes four key metrics in assessing a product: ease of use and retention, accuracy, clinical outcomes, and clinical workflow integration (6). Their guide has several use cases that outline barriers to digital health implementation in specific patient scenarios, and potential solutions where possible. This document serves as a key first step to aid physicians in feeling confident when recommending digital health technologies. This framework does not, however, rectify the need for long term evaluation of patient satisfaction and clinical outcomes in relation to digital health. Guidance documents like these can help direct physicians through the process of incorporating digital health into their practice, but just like the rest of medicine, a comprehensive evidence-based pool of peer-reviewed research will guide practice.

Several groups are beginning to recognize the need for digital health and leveraging technology to substantially advance modern healthcare. Universities in the United States and independent organizations, including the AHA and American Medical Association have set up initiatives and centers for the furthering of digital medicine (23, 47). These programs seek to make medicine more precise and promote integration of medicine and the digital world. The Stanford Center for Digital Health promotes interprofessional collaboration and aid for researching medical technology (48, 49). The Digital Medicine Society is an organization for experts from various fields to aid in the furthering of digital health. They achieve this goal through research, communication and education and community building (49). Assessing the feasibility and clinical efficacy of specific technologies is a cornerstone and foundation to integrating digital health into standard practice across all specialties. By understanding the nuances in measurement, analysis and representation of clinical data in the medical setting requires several parameters to which technology companies along with medical institutions must abide to in concurrence with medical guidelines and scientific society statements. Preventive cardiology continues to be bolstered by the influx of integrative digital health technologies aimed to improve medical monitoring and risk management. Feasibility studies on blood pressure management have shown improvements in patient engagement with BP monitoring in those with acute myocardial infarctions (AMI), as well as those with previous cardiovascular disease and hypertension management post-AMI (50). Validation studies are crucial in not only evaluating the integration of digital health technologies into standard care, but also in assessing the intrinsic validity of measurement tools such as blood pressure and heart rate monitoring devices, where high correlation between manual measurements and wearables shows promising potential particularly in ambulatory medical management (51). Clinical trials and innovative research programs have also become more notable in understanding the holistic impact disruptive technologies such as smart scales measuring fluid and hemodynamic status, AI-based self-management platforms, and smartphone applications, can have in clinical management as well as quality of life in patients with chronic conditions such as heart failure (52). The Connected Health Innovation Research Program (C.H.I.R.P.) started at the Medical College of Wisconsin, extends this growing interest in integrating digital health technologies through research partnerships with innovators, academic institutions, and clinicians to properly assess the utility and adoptability of technologies before integration into cardiovascular care (53). Objective evaluation of parameters such as adoptability and clinical integrity, through retrospective and prospective analysis, play an integral role in confirming adherence to medical guidelines, as well as evaluate the feasibility of introducing new technologies into the traditional model of clinical medicine to improve clinical outcomes through various avenues, not limited to patient education and enhanced patient-physician relationships in the decision-making process (53). Therefore the desire for digital health integration in patient care continues to accelerate through these initiatives and dedicating efforts to research this possibility further.

While we navigate the use of technologies in the medical system, it has become clear that the public is open to their implementation. MSI International, a leading global strategic market research firm, recently found that 4 out of 5 Americans are open to embracing remote monitoring of their health. According to their study of 300 Americans carried out in May 2021, respondents were receptive to allowing physician remote monitoring of their: blood pressure (70%), heart rate (68%), blood sugar (66%) and blood oxygen (65%) (37). This promising result shows that the bottleneck of implementation is most likely specific high quality research guiding physician prescription, recommendation, and insurance coverage of the technologies.

## Complications and barriers of digital health

Digital health, although widely welcomed, is not without barriers or difficulties. Indeed, patients and health care professionals are becoming more comfortable with technology integration into healthcare. However, not all patients will be able to adapt and transition to digital medicine which may at times limit care that deviates from the traditional model of medicine. The World Heart Federation recently released a roadmap to digital health in cardiology (54). In this document several complications and barriers to digital health implementation are discussed. These include health system, health workforce, patient and technological roadblocks (54). The organization also offers solutions to these roadblocks focused on regulation, education and investment in the future of digital health.

We need to be cognizant of potential barriers to adoption of these systems, including perceived usefulness and ease of use from both the physician and patient’s perspectives, design and technical concerns, data privacy concerns, familiarity with the technology, risk-benefit assessment, and communication between health workers and patients (47). Additionally, some patients or patient populations cannot afford costs related to digital health which may not yet be covered by their insurance or may have limited use for certain technologies due to disability (55). While some patients easily accept and appreciate digital health in their standard healthcare, others may be unable to understand and quickly adapt to new technologies. Studies have shown that senior patients, who are often less familiar with newer technologies and yet could sometimes benefit the most, may face difficulty and consequently refuse to use these technologies even if advised (56). Measures will need to be put in place to allocate resources and infrastructure for patient education with these technologies. Optimizing resources, infrastructure, and patient literacy and engagement may help improve adherence, efficacy of technological products, and ultimately improvement in clinical outcomes. The CardioMEMS heart failure system is an applicable example of an effective form of healthcare technology that has yet to be disseminated into everyday healthcare practice despite its evident benefits (22). As previously mentioned, the adoption of digital medicine within everyday practice is influenced by a complex range of factors that stem from not only the technology itself but from healthcare providers, governing institutions and the patients receiving care. Many factors influence consumer use of digital health, including cost of utilization and security concerns that may limit the patient’s eagerness to try new and unfamiliar technology. These factors and others can lead to technologies that have not evolved quickly enough to supply suitable care for a large and medically diverse population. It seems that the most influential factor for the adoption of digital health within everyday healthcare practice is one that we cannot control, which is time itself. The evolution of technology and the growing comfort of this technology with patients and healthcare providers take ample time to ensure long-term implementation into healthcare practice. Despite this revelation, our call to action is for more in depth research that can identify the factors that influence the speed of technology adoption in healthcare to ensure patients receive the most up to date healthcare treatment.

Systems of validation and oversight are needed to ensure health technologies distributed to patient populations collect and provide data that are reliable, accurate, and equitable. Like drug therapies and other types of medical interventions, patient consent in clinical trials evaluating digital health products must employ regulatory processes, ensuring that the safety and health of the patient remain of the utmost priority. Protecting patient privacy is a major barrier to the application of digital health as well. User consent is an ethical concern of digital health as most users do not read the terms of use of the applications (57). Ensuring transparency regarding what data is collected and who can access patient data when taking informed patient consent is a key challenge in digital health adoption (58). The various domains of digital health create data that needs protection, this requires digital health platforms that include anonymization technologies (58). Data breaches can leave patients and institutions vulnerable and can pose major difficulties. The use of multiple digital health technologies could compromise a patient’s protected health information leading to serious consequences such as fraud. Understanding these barriers and complications with incorporating digital health will help reduce patient non-adherence as well as maintain the level of quality healthcare expected in digital health. There needs to be further research into what digital health strategies influence health outcomes and the cost-effectiveness of service delivery (59).

## Learning healthcare system: Data and digital health

### Data sources for learning healthcare systems

A LHS as described by the AHA (5) references four main sources of digital data for the improvement of care: EHR data, clinical registry data, administrative claims data, and supplemental data sources. The AHA outlines the use of EHR, clinical registry, and administrative claims data in detail. The document states that these methods leave out one crucial environment of health: patient health outside of the healthcare delivery setting. Patient health information from outside of the healthcare system arguably represents the majority of potentially actionable data and is a crucial untapped aspect of patient health. Newer data collection technologies such as wearables and implantable trackers provide a more comprehensive view of a patient’s health leading to preventive rather than reactive medicine. One of the greatest challenges outlined by the AHA LHS statement for these technologies is recent naissance and thus lack of standardized methods for analysis and use. Programs that incorporate supplemental data sources help determine their most efficacious uses and viability. While EHR, registry, and claims data will predominate in the near term, they cannot provide a complete perspective on the various factors that influence a patient’s cardiac health; so technologies that g ive an accurate account from outside of hospitals and clinics will increasingly contribute to the cardiovascular learning healthcare system (5). As a result, the learning healthcare system for cardio-oncology will need to develop, integrate, and eventually act on supplemental data sources that can provide critical insights into previously untapped aspects of patient health (5).

### Digital health in learning healthcare systems

LHSs are reliant on four key elements to improve quality and efficiency of health care: science and informatics, patient-clinician partnerships, incentives, and a culture of continuous learning (Figure 1). Digital health in a LHS increases connectivity between the patient and healthcare professionals, providing easier access to information regarding real-world patient physiology, forging a progressive partnership between the patient and clinician and enhancing engagement of patients in their care. Online care delivery platforms such as patient portals can inform, engage, and empower patients in shared decision making to improve autonomy and clinician-patient trust (60). Patient portals give access to online medical consultations, previous after-visit notes, pharmaceutical information, scheduling, and messaging services to connect to their care team for non-emergency questions (60). This gives patients a more prominent role in their care and makes them key contributors to the LHS.

LHS applicable supplemental data sources can be broadly classified as patient-reported data or environmental data (5). Environmental data includes data on patients’ living environments and the impact these environments have on their health (61). Patient-reported data includes information about a patient’s health status (e.g., symptoms, functional status, and quality of life) (61) and physiological measurements (e.g., blood pressure, volume status), which can be collected using traditional methods of inquiry (e.g., questionnaires) or newer data collection methods such as implantable medical devices (e.g., CardioMEMs) and wearables (e.g., FitBit) (5). Collecting and integrating data from these and other domains enables a more comprehensive assessment of an oncology patient’s cardiac health and may improve the ability of the learning healthcare system to proactively anticipate and respond to cardiac health declines or improvements. Supplemental data sources are in their infancy, and additional work is required to develop methods for their collection, analysis, and use (5). Numerous early initiatives that collect and use cardiovascular patient-reported data have demonstrated promise in studies of atrial fibrillation, hypertension, and heart failure (5). For example, studies have shown the accuracy and feasibility of detecting atrial fibrillation and pediatric tachyarrhythmias using sensors integrated into smartphones (32, 62, 63). Studies have also shown promise with remote monitoring of implantable cardioverter-defibrillators and pacemakers (5). This has also been the case with medication adherence (5).

## Disparities and health equity

Racial and ethnic health disparities are prevalent in the United States. These disparities stem from systemic racism and are caused by poor socio-economic outcomes such as decreased financial security, lower educational attainment, and less access to health care. As a result, individuals from marginalized backgrounds bear a greater disease burden. This is apparent in oncology, cardiology, and cardio-oncology, where historically disadvantaged groups experience a higher incidence of cancer occurrence, cardiovascular disease, and cardiotoxicity from cancer therapies (64). African Americans die at a higher rate from cardiovascular disease than non-Hispanic Whites (NHWs) (64). Black women are disproportionately affected by breast cancer mortality and have been shown to be 41% more likely to die of breast cancer than NHW women. (65). Furthermore, Black women with breast cancer are 25% more likely to die from cardiovascular disease than NHW women (66). Even after controlling for cardiovascular risk factors, Black women are twofold more likely to develop cardiotoxicity from trastuzumab use than NHW women (66). Yet, disparities are not limited to the incidence of disease. Risk factors contributing to cardiovascular disease processes weigh more heavily on racial and ethnic minorities. Hypertension has a higher prevalence in African Americans than Caucasians, and is often undertreated in these populations (66, 67). Additionally, African Americans experience earlier onset of diabetes and obesity than Caucasians.

Digital health has the potential to reduce health disparities through the implementation of digital tools in a LHS. Within the LHS, data could be collected about user access to healthcare with metrics like broadband access and visit satisfaction for virtual visits. The LHS could be used to track the impact of improving access to digital tools for underserved groups. Digital tools like “hot-spotting” could also be used to identify underserved healthcare areas where additional resources or programming can be applied to impact social determinants of health, which could contribute heavily to the prevention of disease. Underserved patients with conditions that can be managed with remote monitoring could also receive both technology and education on how to use these tools. Further, modern communication technologies could be used to distribute educational materials specific to these patients to improve engagement. A LHS can use digital meeting rooms to facilitate new community group meetings or to allow individuals to attend existing meetings remotely to make attendance more convenient and improve participation in the patient-provider dialogue described in the AHA learning healthcare system statement (5).

Through the incorporation of digital health tools into the LHS, health disparities can be further studied through clinical research. As we incorporate digital health tools into the LHS, it is appropriate to leverage these tools to better understand racial and ethnic health disparities through clinical research. This research must focus on equitable representation of racial and ethnic minorities in clinical research, such as incorporating data from diverse populations in pre-clinical studies and increasing marginalized group participation in clinical trials (64). Engaging marginalized groups in clinical research through digital health tools is promising, as these tools are being used by racial and ethnic minorities (17). For example, there was increased usage of telemedicine services among African Americans and other marginalized groups during the COVID-19 pandemic (68). However, marginalized groups still face several challenges with respect to using digital health tools such as lower health literacy and reduced internet access and conducting digital health research and incorporating digital health interventions into the care of these groups should focus on increasing accessibility (68). Clearly more than just incorporation of digital tools into the LHS and clinical research engagement is needed to address the health disparities that racial and ethnic minorities face. Efforts need to be made to dismantle structural racism, engage the community, and improve access to cardio-oncology services (64).

## Conclusion

Digital health, in many present and future forms, continues to become increasingly relevant in modern medicine. By understanding the importance, capabilities, and limitations of digital health, innovators in healthcare can continue working toward refining how we utilize technology in medicine. In addition, as we have seen in the past year, telemedicine and digital health have the opportunity to provide an economical alternative to providing quality care with increased access to many patient populations (16, 17, 32, 68, 69). With this understanding, we can recognize that digital health will remain an integral aspect of the new era in medicine and the successful implementation of a LHS as described by the AHA.

While digital data are already highly integrated into the EHR, clinical registry, and administrative claims branches of the LHS data systems, digital health has enormous potential to mold the supplementary data branch by providing reports on patient health outside the healthcare setting. The production and research of digital health tools will revolutionize the healthcare system and the quality of care we can provide. While digital health tools continue to be developed and show promise, they outpace the rate that research on the efficacy of these tools can be conducted. Indeed, studies have shown that digital health interventions can improve medication adherence, provide remote data to inform accurate diagnoses and monitor pre-pathological states, among other benefits. However, more steps must be taken to implement digital health interventions into evidence-based clinical practice. To make this a reality in modern patient care, providers must feel comfortable recommending these technologies and guidelines, such as the ACC Best Practices for Consumer Cardiovascular Technology Solutions (8, 9). In addition, design concerns, such as providing appropriate patient education, and technical concerns, such as data breaches, must be addressed. Research must be conducted on the benefits and risks of digital health interventions before widespread implementation. Although this field of study is still in its early stages, we have an advantage in terms of designing studies to accurately represent the population and determine optimal ways to increase access to combat racial and ethnic disparities.

## Author contributions

RM and SAB: conception and design. RM, JM, TM, SP, AS, GB, AH, JW, and SAB: drafting of the manuscript. RM, JM, TM, SP, AS, GB, AH, and SAB: interpretation of data. RM, JM, TM, SP, AS, GB, AH, MA, JW, and SAB: critical revision. All authors have read and approved the final manuscript.
